# Safety, tolerability, and pharmacokinetics of ibrexafungerp in healthy Chinese subjects: a randomized, double-blind, placebo-controlled phase 1 trial

**DOI:** 10.1128/aac.01075-23

**Published:** 2023-11-16

**Authors:** Xiaoyan Liu, Rui Zhang, Rong Li, Qiong Wu, Chao Pan, Xiangqing Yu, Yuhui Liu, Benjie Wang, Shuwen Yu

**Affiliations:** 1 Phase 1 clinical Trial Center, Qilu Hospital of Shandong University, Jinan, China; 2 NMPA Key Laboratory for Clinical Research and Evaluation of Innovative Drugs, Shandong University, Jinan, China; 3 Jiangsu Hansoh Pharmaceutical Group Co. Ltd., Lianyungang, China; Providence Portland Medical Center, Portland, Oregon, USA

**Keywords:** ibrexafungerp, HS-10366, fungal infection, phase 1, pharmacokinetics, Chinese

## Abstract

Ibrexafungerp (code name in China: HS-10366) is a first-in-class and orally active triterpenoid antifungal agent with broad antifungal activity against *Candida* spp., *Aspergillus* spp., and other fungal pathogens. It was approved by the U.S. Food and Drug Administration for the treatment of vulvovaginal candidiasis. The study aimed to evaluate the safety, tolerability, and pharmacokinetic (PK) characteristics of oral ibrexafungerp in healthy Chinese adults. A single-center, randomized, double-blind, placebo-controlled single ascending dose (SAD, *n* = 42), and multiple ascending dose (MAD, *n* = 28) study was conducted in healthy Chinese subjects from March to October 2022. There were three cohorts in the SAD stage (300, 600, and 1,500 mg) and two cohorts in the MAD stage [450 mg once daily (QD) for 7 days; a loading dose of 750 mg twice daily (BID) for the first 2 days followed by a maintenance dose of 750 mg QD for consecutive 5 days]. Eligible participants in each cohort were randomly assigned in a 6:1 ratio to receive either ibrexafungerp or placebo orally. The primary objectives were to evaluate the safety and tolerability. The secondary objective was to evaluate PK parameters, including C_max_, AUC, and t_1/2_. A total of 70 healthy Chinese subjects were enrolled in the study. The mean (SD) age was 29.0 (6.32), and 55.7% were male. All treatment-emergent adverse events (TEAEs) were mild or moderate. There were no serious adverse events, and no subjects were discontinued from the study due to TEAEs. All TEAEs were recovered or resolved. The most common TEAEs were diarrhea, abdominal pain, and nausea. In the SAD stage, C_max_, and AUC increased in an approximately dose-proportional manner in the dose range of 300–1,500 mg. The mean t_1/2_ was within 18.29–21.30 hours. In the MAD stage, an accumulation of exposure (C_max_ and AUC) was observed following multiple doses. This phase 1 study demonstrates a favorable safety, tolerability, and PK profile of ibrexafungerp in healthy Chinese subjects.

## INTRODUCTION

*Candida* species, the notable fungal pathogen, are the most common cause of invasive fungal infection (1, 2). Worldwide, infections from *Candida* species lead to prolonged hospitalization, substantial mortality, and increased healthcare costs, especially for critically ill and immunosuppressed patients (3, 4). During the past several decades, echinocandins have emerged as first-line antifungal agents for many *Candida* infections and have a promising efficacy and favorable safety profile. However, there is increasing evidence of development of resistance to echinocandins in multiple *Candida* species, especially in *C. glabrata*, over recent years (5). In addition, intravenous-only administration of echinocandins significantly limits the clinical use of these agents. Azoles and polyenes are two classes of second-line antifungal agents for the treatment of invasive candidiasis (6, 7). These two classes of agents exhibit several limitations, including increased drug-resistance, hepatic toxicity, and drug-drug interaction liability (i.e., fluconazole); serious nephrotoxicity, poor oral bioavailability and a potential for causing hypokalemia in non-liposomal amphotericin B (8–10). Therefore, it is urgent to develop novel antifungal treatments to overcome these serious challenges.

Ibrexafungerp (formerly SCY-078 or MK-3118), code name HS-10366 in this study, is a novel triterpenoid antifungal agent in development for the treatment of fungal infection (11). It targets the glucan synthase resulting in fungal cell death, which is also the mechanism of echinocandins. However, compared to echinocandins, ibrexafungerp has demonstrated the advantages of oral bioavailability, wide tissue distribution, excellent tissue penetration, and maintains active to the majority of echinocandin-resistant *Candida* species (12). A significant elimination path for ibrexafungerp is metabolizing by CYP3A4, followed by glucuronidation and sulfation of a hydroxylated inactive metabolite. Based on the positive results from three pivotal phase 3 studies [VANISH 303 (NCT03734991), VANISH 306 (NCT03987620), and CANDLE (NCT04029116)] (13–15), Ibrexafungerp has been approved by the FDA for the treatment of adult and post-menarchal pediatric females with vulvovaginal candidiasis in June 2021, and for the reduction in the incidence of recurrent vulvovaginal candidiasis in December 2022, respectively (16, 17). The recommended dosage regimen is an oral administration of 300 mg approximately every 12 hours for one day, or every 12 hours for one month with a consecutive up to six months, respectively.

Ethnic factor is important in explaining variability in drug safety, efficacy, and dosage among individuals from different regions (18, 19). The International Council for Harmonization (ICH) accordingly published guidance to recommend a framework for evaluating the impact of ethnic factors before the registration of a drug in a new region. Nevertheless, the majority of clinical studies for ibrexafungerp were conducted in Western countries, and most subjects enrolled in these trials were non-Asian. Goje and colleagues reported no differences in ibrexafungerp efficacy between White and Black patients, based on the pooled data of two phase 3 studies, VANISH 303 and VANISH 306 (20). Due to the limited number of Asian patients in these two studies, pharmacological differences between Chinese and non-Asian populations could not be determined.

Therefore, the first-in-Chinese phase 1 clinical trial of ibrexafungerp has been conducted, and a pivotal phase 3 clinical trial (http://www.chinadrugtrials.org.cn; identifiers are CTR20220919 and CTR20220918, respectively) in the Chinese population has been completed. Herein, we reported the results of the phase 1 study to evaluate the safety, tolerability, and pharmacokinetics (PK) of single and multiple ascending oral doses of ibrexafungerp in healthy Chinese subjects.

(The data in this manuscript were partly presented in abstract/poster form at the Infectious Disease Week (ID Week), 11 to 15 October 2023, Boston, Massachusetts.)

## MATERIALS AND METHODS

### Study design

This was a randomized, double-blind, placebo-controlled phase 1 study, aiming at assessing the safety, tolerability, and PK characteristics of ibrexafungerp in healthy Chinese subjects. Two stages, the single ascending dose (SAD) stage and the multiple ascending dose (MAD) stage, were launched sequentially.

This study was conducted in the Qilu Hospital of Shandong University from 3 March to 9 October 2022 in line with ethical principles derived from the Declaration of Helsinki and in compliance with the International Council for Harmonization Guidelines for Good Clinical Practice. The protocol, informed consent form (ICF), recruitment materials, all subject materials, and any amendments were submitted and approved by the Ethics Committee of Qilu Hospital of Shandong University. Written informed consent was obtained before the implementation of any study-related procedures.

**Fig 1:**
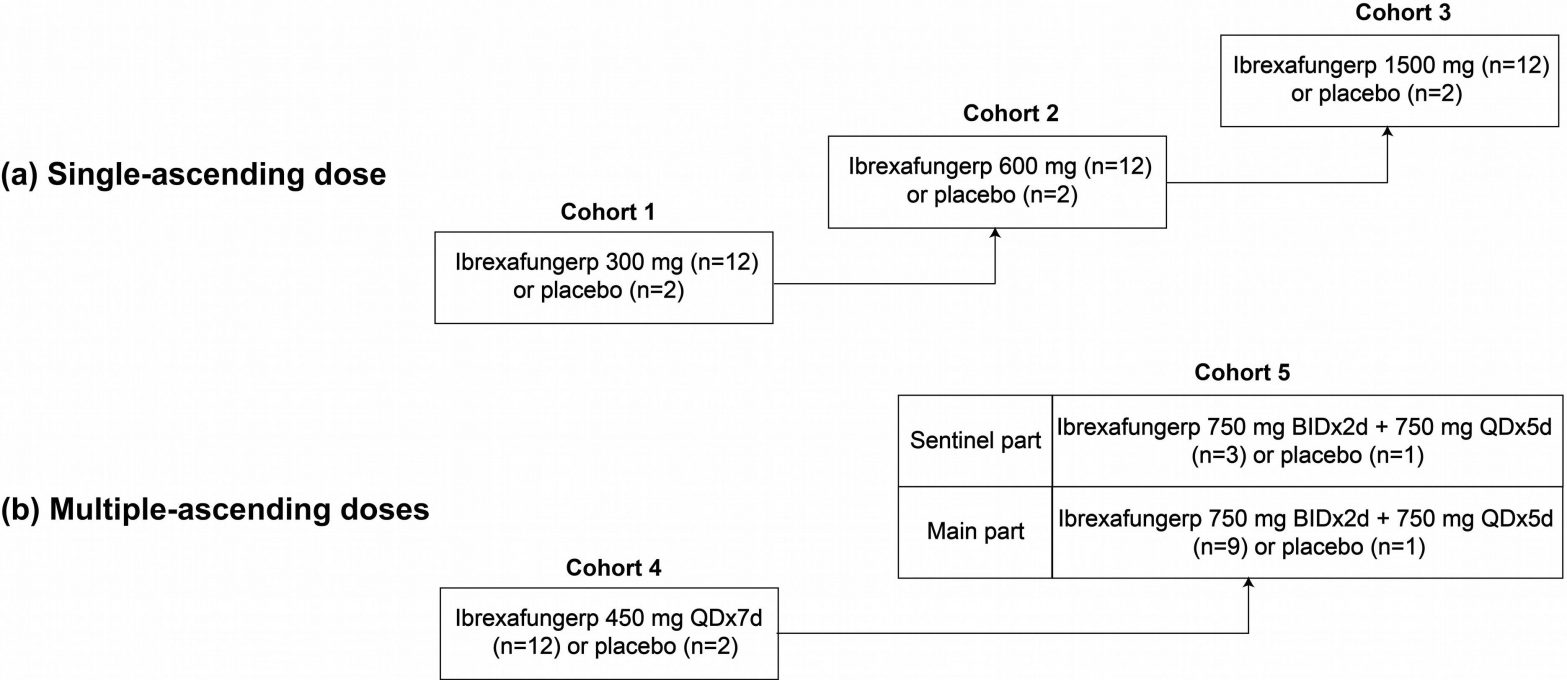
Overall design, treatments, and numbers of subjects enrolled in single-ascending dose (**a**) and multiple-ascending dose (**b**)of this first-in-Chinese study. In this study, which was conducted at Phase 1 Clinical Trial Center of Qilu Hospital, ibrexafungerp was given as an oral citrate tablet. Unless otherwise specified, ibrexafungerp was administered under fasting conditions excepted for subjects in cohort 5, which were given a standard meal. QD, once daily; BID, twice daily.

Eligible subjects were randomly assigned in a 6:1 ratio to receive an ibrexafungerp citrate tablet (strengths: 150 mg and 250 mg) or matching placebo (Fig. 1). A total of 70 healthy subjects, including 42 in the SAD stage (cohorts 1, 2, and 3; *n* = 14 for each cohort; Fig. 1a) and 28 in the MAD stage (cohorts 4 and 5; *n* = 14 for each cohort; Fig. 1b), were enrolled to the study. Single doses of 300, 600, and 1,500 mg ibrexafungerp or placebo were administered in cohorts 1, 2, and 3 in the SAD stage, respectively. The multi-dosing schedule of ibrexafungerp or placebo in cohort 4 in the MAD stage was 450 mg once daily (QD) for 7 days. For the higher dose cohort (cohort 5) in the MAD stage, a sentinel sub-cohort design was utilized to prevent unexpected serious adverse events occurring in the entire cohort. In short, cohort 5 of the MAD stage contained a sentinel part (ibrexafungerp versus placebo = 3:1) and a main part (ibrexafungerp versus placebo = 9:1), with a loading dose of ibrexafungerp or placebo of 750 mg twice daily (administered every 12 hours, Q12H) for the first 2 days followed by a maintenance dose of 750 mg QD for the following consecutive 5 days. The study procedures and contents in the sentinel part of cohort 5 were consistent with the main part except that subjects in the main part started four days later than the sentinel part. From cohort 1 to cohort 4, ibrexafungerp was administered during fasting, while ibrexafungerp was given with a standard meal in cohort 5.

Eligible subjects included in this study were healthy volunteers, male or female (those who were not in pregnancy or lactating), aged between 18 and 45 years old, with a body mass index (BMI) between 19 and 27 kg/m^2^, and body weight ≥50 kg for males and ≥45 kg for females at screening. Consumption of foods that affect drug metabolism (such as grapefruit, blood oranges, vegetables from the mustard green family, and charbroiled meats) within 2 weeks prior to screening was prohibited. Subjects with a history or presence of any condition that deemed by the investigator would interfere with the study were excluded. Subjects who have a known hypersensitivity to ibrexafungerp or any of the components of the formulation were also excluded. The detailed criteria for inclusion, exclusion, and stopping dose escalation were summarized in the Supplementary material. For each dose escalation or bridging decision, preliminary cumulative safety, tolerability, and PK parameters from previous cohorts were evaluated.

### Safety and tolerability assessments

Safety and tolerability assessments included adverse events (AEs) and serious AEs (SAEs) assessments from the signing of ICF until finishing assessments in the last visit of follow-up. Time intervals between the last dose and last visit of follow-up in SAD and MAD stages were both 7 days. Severity of AEs was classified mainly according to the protocol of this trial, CTCAE (version 5.0) was also referred to verify the judgment. AEs were coded by the Medical Dictionary for Regulatory Activities (MedDRA) version 25.1 term (MedDRA MSSO, Herndon, VA, http://www.meddra.org). Vital signs, physical examinations, 12-lead electrocardiograms (ECGs), and laboratory tests were monitored closely at each prespecified time point according to our protocol.

### Sample collection and bioanalytical assay

In all three cohorts of the SAD stage, blood samples for ibrexafungerp concentration were collected predose (0 hour), and at 0.5, 1, 1.5, 2, 3, 4, 5, 6, 8, 12, 16, 24, 36, 48, 72, and 96 hours postdose. In 450 mg cohort of MAD stage, blood samples were collected predose (0 hour), and at 0.5, 1, 1.5, 2, 4, 6, 8, 12, and 24 hours postdose on day 1; predose (0 hour) from day 5 to day 7; at 0.5, 1, 2, 4, 6, 8, 12, 24, 48, 72, and 96 hours postdose on day 7. In 750 mg cohort of MAD stage, blood samples were collected predose (0 hour), and at 2, 4, 6, and 8 hours postdose in the morning and evening on day 1; predose (0 hour) from day 2 to day 6; predose (0 hour), and 2, 4, 6, 8, 12, 16, 24, 36, 48, 72, 96, and 120 hours postdose on day 7.

A validated HPLC-MS/MS assay was used to quantify the concentrations of ibrexafungerp in human plasma. Samples were pretreated by protein precipitation extraction. The calibration range was 5.00–5,000 ng/mL. The sensitivity of this method was 5.00 ng/mL. The maximum of intra-run and inter-run precision (CV%) were 5.27 and 4.32, respectively. The range of intra-run and inter-run accuracy (Bias%) were −0.67 ~ 11.50 and 1.44 ~ 9.07, separately. Moreover, matrix effect, selectivity, dilution, carryover, and stability were also validated. All performance characteristics of the bioanalytical method met the acceptable requirement. All samples were collected, stored, and analyzed under conditions confirmed by analyte stability during validation studies.

### Pharmacokinetic data analysis

PK data analyses were performed using the data from all enrolled subjects who had at least one plasma concentration post-dosing. PK parameters for ibrexafungerp were calculated with standard non-compartmental analysis using Phoenix WinNonlin (Certara USA, Inc., version 8.1) and were summarized by study part and cohort using SAS (version 9.4, SAS Institute Inc., NC, USA). Plasma concentrations of HS-10366 were summarized and plotted using Phoenix WinNonlin (Certara USA, Inc., version 8.1).

### Statistical analysis

All analyses were performed by using SAS software (version 9.4, SAS Institute Inc., NC, USA) or Phoenix WinNonlin (version 8.0, Certara USA Inc., USA). Demographics and baseline characteristics as well as safety data were summarized descriptively for subjects who had received at least one dose of study drug. Mean (standard deviation) and count (percentage) were presented for continuous data and categorical data, respectively.

## RESULTS

### Demographic profile

In total, 70 subjects were randomized into this study (42 in the SAD stage and 28 in the MAD stage). For both stages, all subjects are Chinese with balanced distributions of age, sex, ethnicity, and BMI between ibrexafungerp and placebo groups (Tables 1 and 2). In the SAD stage, the mean age of ibrexafungerp and placebo groups was 27.9 and 33.2 years; the mean BMI of two groups was 22.26 and 23.20 kg/m^2^, respectively (Table 1). In the MAD stage, the mean age of ibrexafungerp and placebo groups was 29.2 and 31.3 years; the mean BMI of two groups was 23.15 and 23.65 kg/m^2^, respectively (Table 2). The demographic and baseline characteristics of the study population in SAD and MAD stages were presented in Tables 1 and 2. Of the randomized 70 subjects, 67 (39 in the SAD stage and 28 in the MAD stage) completed the study. Three subjects were lost to follow-up, and all of the three subjects were in the SAD stage (two in placebo group and one in ibrexafungerp group of 1,500 mg cohort).

**TABLE 1:** Demographic and clinical characteristics of subjects in the single ascending dose (SAD) stage

	Placebo (*n* = 6)	Ibrexafungerp		
		300 mg (*n* = 12)	600 mg (*n* = 12)	1,500 mg (*n* = 12)
Age (years)				
Mean (SD)	33.2 (9.06)	28.3 (7.55)	26.0 (4.69)	29.3 (6.30)
Sex, *n* (%)				
Male	2 (33.3)	7 (58.3)	8 (66.7)	6 (50.0)
Female	4 (66.7)	5 (41.7)	4 (33.3)	6 (50.0)
Ethnicity, *n* (%)				
Han	6 (100.0)	12 (100.0)	12 (100.0)	11 (91.7)
Others	0	0	0	1 (8.3)
Height (cm)				
Mean (SD)	161.00 (9.252)	165.33 (8.195)	167.92 (5.760)	164.08 (8.129)
Weight (kg)				
Mean (SD)	60.50 (11.178)	64.15 (7.788)	61.49 (7.647)	58.13 (7.480)
BMI (kg/m^2^)				
Mean (SD)	23.20 (2.439)	23.44 (1.953)	21.76 (1.972)	21.57 (1.932)

**TABLE 2:** Demographic and clinical characteristics of subjects in the multiple ascending dose (MAD) stage

	Placebo (*n* = 4)	Ibrexafungerp	
		450 mg (*n* = 12)	750 mg (*n* = 12)
Age (years)			
Mean (SD)	31.3 (4.27)	29.1 (5.96)	29.3 (5.65)
Sex, *n* (%)			
Male	2 (50.0)	7 (58.3)	7 (58.3)
Female	2 (50.0)	5 (41.7)	5 (41.7)
Ethnicity, *n* (%)			
Han	4 (100.0)	11 (91.7)	12 (100.0)
Others	0	1 (8.3)	0
Height (cm)			
Mean (SD)	167.13 (13.181)	167.71 (9.396)	165.88 (9.521)
Weight (kg)			
Mean (SD)	66.23 (9.992)	64.59 (8.989)	64.74 (11.476)
BMI (kg/m2)			
Mean (SD)	23.65 (1.145)	22.94 (2.503)	23.37 (2.179)

### Safety and tolerability

All 70 subjects received at least one dose of study drug and were included in the safety analysis sets. TEAEs were reported in 8 (66.7%), 11 (91.7%), 12 (100.0%), 12 (100.0%), and 12 (100.0%) subjects in SAD and MAD stages. All TEAEs were mild to moderate in severity. There were no SAEs, severe AEs, or AEs leading to withdrawal during this study. The most common TEAEs in SAD and MAD stages was diarrhea (69.4% and 91.7%, respectively). The most common ibrexafungerp-related TEAEs were also the most common TEAEs. The comparative analysis between SAD and MAD stages showed that the incidence of diarrhea gradually increased with increasing doses. Most TEAEs were resolved without corrective interventions except for three reports (two reports of abdominal pain and one report of syncope) from three subjects in 1,500 mg cohort, who recovered after being treated with an infusion of electrolyte balance solutions and anisodamine. All ibrexafungerp-related TEAEs in SAD and MAD stages were presented in Tables 3 and 4. In addition, all TEAEs of SAD and MAD stages were shown in Supplemental material.

**TABLE 3:** Study drug-related TEAEs in the single ascending dose (SAD) stage

	Placebo (*n* = 6) N (%)	Ibrexafungerp		
		300 mg (*n* = 12) N (%)	600 mg (*n* = 12) N (%)	1,500 mg (*n* = 12) N (%)
Study drug-related TEAEs	3 (50.0)	6 (50.0)	9 (75.0)	12 (100.0)
Gastrointestinal disorders	1 (16.7)	5 (41.7)	8 (66.7)	12 (100.0)
Diarrhea,	1 (16.7)	5 (41.7)	8 (66.7)	12 (100.0)
Abdominal pain	0	5 (41.7)	1 (8.3)	7 (58.3)
Vomiting	0	0	0	4 (33.3)
Nausea	0	0	0	3 (25.0)
Abdominal bloating	0	0	1 (8.3)	0
Laboratory abnormalities	1 (16.7)	2 (16.7)	5 (41.7)	6 (50.0)
Hemoglobin decreased	0	0	2 (16.7)	2 (16.7)
Blood triglycerides increased	1 (16.7)	2 (16.7)	0	1 (8.3)
Urine erythrocyte positive	0	0	1 (8.3)	1 (8.3)
Urine occult blood positive	0	0	0	2 (16.7)
Presence of urinary ketones	0	0	0	2 (16.7)
Uric acid increased	0	0	1 (8.3)	1 (8.3)
LDL increased	0	0	0	1 (8.3)
Prolonged thrombin time	0	0	0	1 (8.3)
ECG ST segment elevated	0	0	1 (8.3)	0
Abnormal ECG T-wave	0	1 (8.3)	0	0
Blood bilirubin increased	0	0	1 (8.3)	0
Vascular and lymphatic diseases	1 (16.7)	2 (16.7)	2 (16.7)	0
Orthostatic hypotension	1 (16.7)	2 (16.7)	2 (16.7)	0
Neurological diseases	1 (16.7)	0	0	1 (8.3)
Dizziness	0	0	0	1 (8.3)
Syncope	1 (16.7)	0	0	1 (8.3)
Heart diseases	1 (16.7)	0	1 (8.3)	0
Sinoatrial block	0	0	1 (8.3)	0
Ventricular extrasystole	1 (16.7)	0	0	0

**TABLE 4:** Study drug-related TEAEs in the multiple ascending dose (MAD) stage^*a*^

	Placebo (*n* = 4) N (%)	Ibrexafungerp	
		450 mg, (*n* = 12) N (%)	750 mg, (*n* = 12) N (%)
Study drug-related TEAEs	2 (50.0)	12 (100.0)	12 (100.0)
Gastrointestinal disorders	1 (25.0)	10 (83.3)	12 (100.0)
Diarrhea	0	10 (83.3)	12 (100.0)
Abdominal pain	1 (25.0)	5 (41.7)	10 (83.3)
Nausea	0	2 (16.7)	6 (50.0)
Vomiting	0	0	6 (50.0)
Gastroesophageal reflux disease	0	0	2 (16.7)
Constipation	0	1 (8.3)	0
Abdominal bloating	0	0	1 (8.3)
Oral mucositis	0	0	1 (8.3)
Laboratory abnormalities	1 (25.0)	5 (41.7)	7 (58.3)
Blood bilirubin increased	0	2 (16.7)	3 (25.0)
Alanine aminotransferase increased	0	3 (25.0)	0
Blood pressure increased	0	1 (8.3)	2 (16.7)
Urine occult blood positive	0	0	2 (16.7)
Aspartate aminotransferase increased	0	2 (16.7)	0
Blood triglyceride increased	1 (25.0)	2 (16.7)	0
Blood creatine kinase increased	0	0	1 (8.3)
Metabolic and nutritional diseases	0	1 (8.3)	3 (25.0)
Hypertriglyceridemia	0	0	3 (25.0)
Hyperglycemia	0	1 (8.3)	0
Heart diseases	0	1 (8.3)	2 (16.7)
Tachycardia	0	1 (8.3)	2 (16.7)
Infectious and infective diseases	0	0	2 (16.7)
Urinary tract infection	0	0	2 (16.7)
Neurological diseases	0	0	2 (16.7)
Dizziness	0	0	2 (16.7)
Systemic disease and various reactions at the site of administration	0	1 (8.3)	0
Chest discomfort	0	1 (8.3)	0

^*a*^ Note: The percentage was calculated using the number of subjects in each cohort of the safety analysis set as the denominator. Adverse events associated with the study drug were defined as those in which the association with the study drug during administration was “definitely relevant”, “probably relevant”, or “undetermined”. Adverse events were coded based on MedDRA 25.1.

### Pharmacokinetic properties

Plasma concentrations of ibrexafungerp were available for analysis in 60 healthy Chinese subjects (36 in the SAD stage and 24 in the MAD stage), and these 60 subjects were included in the PK analysis.

**Fig 2:**
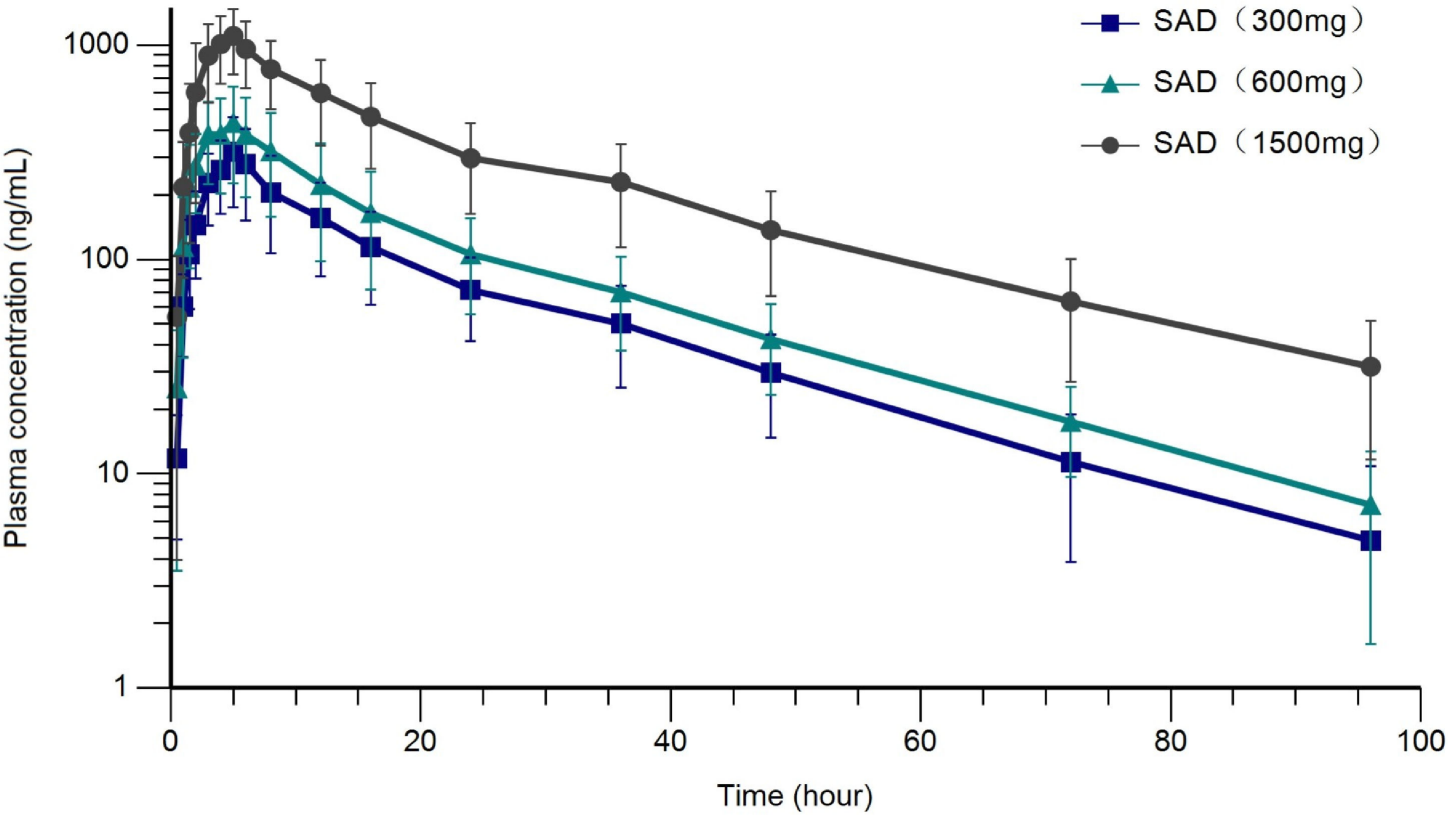
Plasma ibrexafungerp concentration (Mean ± SD, log plots) time profiles after a single dose. SAD, single ascending dose.

After a single oral dose in the fasting state, the mean plasma concentration of ibrexafungerp generally increased with increasing dose in a dose range of 300 to 1,500 mg (Fig. 2). After a single oral dose of ibrexafungerp, the median T_max_ ranged from 4.0 to 5.0 hours. The mean t_1/2_ was estimated to be approximately 18.29–21.30 hours. The ranges of mean CL/F and V_d_/F were 57.68–77.47 L/h and 1521.88–2216.81 L, respectively. The large Vd/F suggested a wide distribution in human tissues. Mean λ_z_, t_1/2_, V_d_/F, CL/F, and MRT were generally consistent across the dose level (Table 5). The estimated slopes of the power model for C_max_ (slope = 0.83, 90% CI: 0.64–1.02), AUC_0−t_ (slope = 0.91, 90% CI: 0.71–1.11), and AUC_0−∞_ (slope = 0.91, 90% CI: 0.71–1.10) were all less than one after a single dose. The estimated slope for AUC was close to 1, suggesting that exposure was approximately dose-proportional.

**TABLE 5:** Descriptive summary of pharmacokinetic parameters of ibrexafungerp in the single ascending dose (SAD) stage (fasting)^*b*^

Parameter (unit)	300 mg *n* = 12	600 mg *n* = 12	1,500 mg *n* = 12
C_max_ (ng/mL)^*c*^	291.69 (48.03)	425.13 (47.21)	1086.01 (41.73)
T_max_ (h)^*a*^	5.0 (3.0, 5.0)	4.0 (2.0, 6.0)	5.0 (2.0, 12.0)
AUC_0−t_ (h·ng/mL)	4936.07 (52.79)	7437.71 (47.32)	21030.89 (46.02)
AUC_0−∞_ (h·ng/mL)	5201.03 (50.74)	7744.98 (45.60)	21905.80 (47.12)
t_1/2_ (h)	18.29 (21.03)	19.84 (20.84)	21.30 (15.55)
CL/F(L/h)	57.68 (50.74)	77.47 (45.60)	68.48 (47.12)
V_d_/F (L)	1521.88 (43.20)	2216.81 (59.67)	2104.54 (41.49)
λ_z_ (1 /h)	0.04 (21.03)	0.04 (20.84)	0.03 (15.55)
MRT (h)	24.06 (19.82)	24.57 (20.56)	27.36 (16.48)

^*a*^ Median (minimum, maximum).

^*b*^ Note: Parameters were shown as Geometric Mean (coefficient of variability CV %), unless otherwise noted.

^*c*^ Abbreviations: *n*, Number of subjects; C_max_, Maximum observed plasma concentration; T_max_, Time of C_max_; AUC_0−t_, Area under the concentration-time curve from time 0 to the last quantifiable time; AUC_0−∞_, Area under the concentration-time curve from time 0 extrapolate to infinity; t_1/2_, Terminal half-life; CL/F, Apparent clearance; Vd/F, Apparent volume of distribution; λz, Terminal rate constant; MRT, Mean residence time.

**Fig 3:**
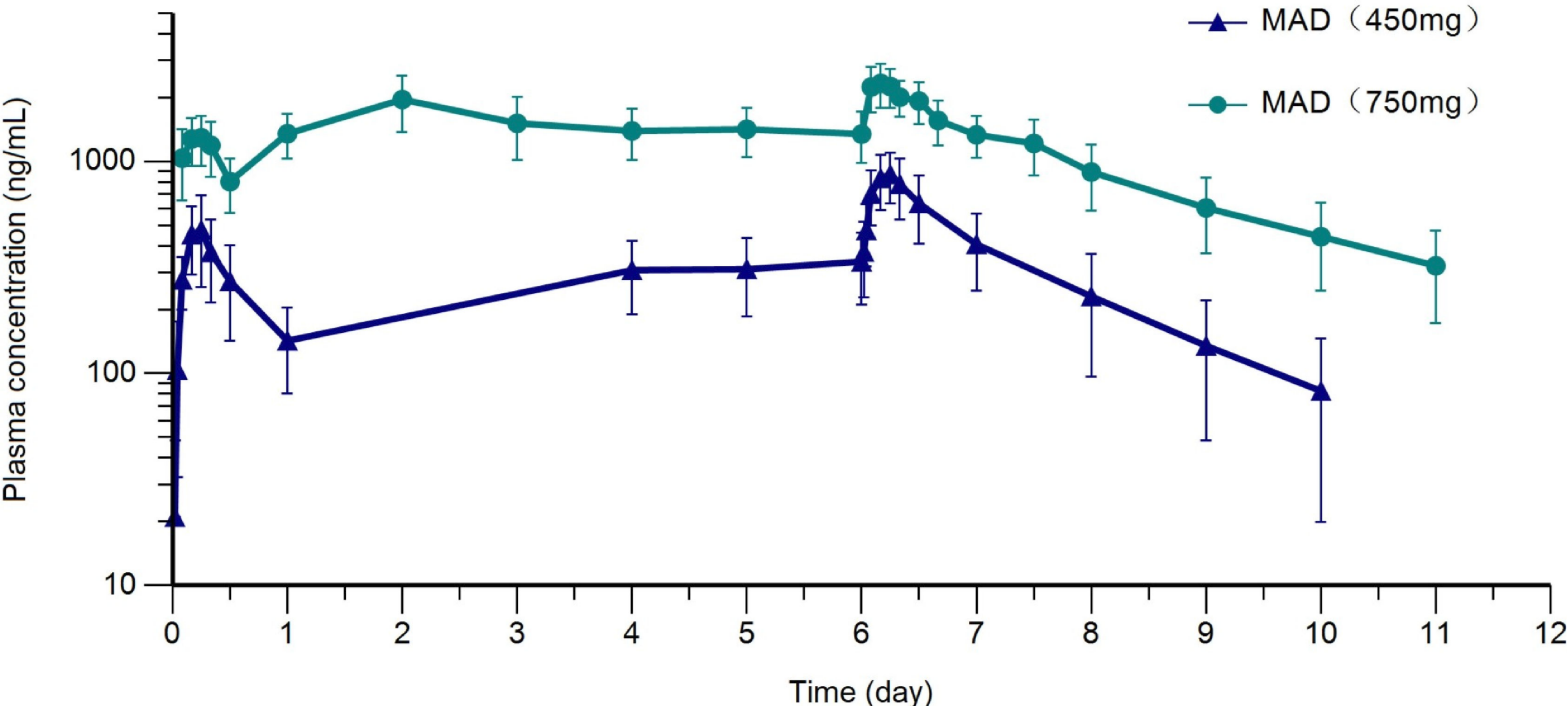
Plasma ibrexafungerp concentration (Mean ± SD, log plots)-time curve after repeated doses. MAD, multiple ascending doses.

After multiple doses of 450 mg (fasting) or 750 mg (taken with a standard meal), mean trough plasma concentrations plateaued on days 5, 6, and 7, indicating reaching a steady state (Fig. 3). The median T_ss,max_ was 6.0 and 4.0 hours for the 450 mg and the 750 mg cohort, respectively. Steady-state exposure (C_ss,max_ and AUC_ss_) after multiple doses of 450–750 mg increased in a more than dose-proportional manner, which might be in part associated with a food effect in 750 mg cohort. The mean accumulation ratios of C_max_ and AUC were in a range of 1.78 and 2.34, indicating a mild to moderate exposure accumulation (Table 6). Overall, the inter-subject variability in PK parameters of ibrexafungerp was mild to moderate in the current study.

**TABLE 6:** Descriptive summary of pharmacokinetic parameters of ibrexafungerp in the multiple ascending dose (MAD) stage^*b*^

Parameter (unit)		450 mg (fasting) *n* = 12	750 mg (with a standard meal) *n* = 12
Day 1	C_max_ (ng/mL)^*c*^	463.78 (40.65)	1301.00 (24.29)
	T_max_ (h)^*a*^	4.0 (2.0, 6.0)	6.0 (4.0, 6.0)
	AUC_0−τ_ (h·ng/mL)	6109.46 (37.33)	10905.92 (23.69)
Day 7	C_ss,max_ (ng/mL)	854.67 (25.96)	2320.17 (22.56)
	T_ss,max_ (h)^*a*^	6.0 (2.0, 8.0)	4.0 (2.0, 4.0)
	AUC_ss_ (h·ng/mL)	14291.85 (30.08)	42287.78 (24.54)
	CL_ss_/F (L/h)	31.49 (30.08)	17.74 (24.54)
	V_ss_/F (L)	1368.85 (24.53)	1164.89 (21.10)
	R_cmax_	1.84 (48.15)	1.78 (19.04）
	R_auc_	2.34 (45.35)	2.23 (17.19)

^*a*^ Median (minimum, maximum).

^*b*^ Note: Parameters were shown as Geometric Mean (coefficient of variability CV %), unless otherwise noted.

^*c*^ Abbreviations: *n*, Number of subjects; C_max_, Maximum observed plasma concentration; T_max_, Time of C_max_; AUC_0, τ_, Area under the concentration-time curve during dosing interval (the dosing interval of 450 mg cohort and 750 mg cohort is 24 and 12 hours in day 1, respectively), C_ss, max_, The maximum concentration at steady state; T_ss, max_, Time of the maximum concentration at steady state; AUC_ss_, Area under the concentration-time curve at steady state; CL_ss_/F, Apparent clearance at steady state; V_ss_/F, Apparent volume of distribution at steady state; R_cmax_, Accumulation ratio of C_max_; R_auc_, Accumulation ratio of AUC (for 750 mg cohort, R_auc_ = AUC_0−12_, day7/AUC_0−τ_).

## DISCUSSION

This was the first study to assess the safety, tolerability, and PK of ibrexafungerp in healthy Chinese subjects. In the SAD stage, C_max_, and AUC of ibrexafungerp increased in an approximately dose-proportional manner in the range of 300 to 1,500 mg. The median T_max_ and mean t_1/2_ were similar across all doses. In the MAD stage, the mean accumulation ratios of AUC and C_max_ ranged from 1.78 to 2.34, indicating a mild to moderate accumulation of ibrexafungerp after repeated doses.

With respect to the safety of ibrexafungerp in healthy Chinese subjects, most TEAEs were mild to moderate, and resolved without medical interventions. The most common TEAEs in SAD and MAD stages were gastrointestinal disorders (including diarrhea, abdominal pain, and vomiting), which were similar to that in non-Asian populations. Moreover, no evidence of gastrointestinal degeneration or any significant clinical abnormalities was found in any biopsy of subjects in phase 1 studies overseas. These data indicated that the effect of ibrexafungerp on gastrointestinal mucosa was without significant functional or safety consequences and was completely reversible in humans.

With access to two previous phase 1 studies overseas (FDA application review files of ibrexafungerp), the PK characteristics of ibrexafungerp could be compared between Asian and non-Asian subjects. In the present study, C_max_ and AUC_0**−∞**_ of HS-10366 were 425.13 ng/mL and 7,744.98 h·ng/mL, respectively in the 600 mg cohort after overnight fasting. While the C_max_ and AUC_0**−∞**_ of ibrexafungerp were 459.13 ng/mL and 11,734.82 h·ng/mL in the previous study and the same 600 mg ibrexafungerp doses were administrated after overnight fasting. Obviously, mean C_max_ values were similar between studies, but the AUC_0**−∞**_ of ibrexafungerp in healthy Chinese subjects was lower than that in non-Asian subjects. Meanwhile, the measured t_1/2_ in the healthy Chinese subjects was comparable in healthy non-Asian populations (19.84 vs. 18.70 hours). Accumulation of ibrexafungerp after continuous dosing (750 mg BID for the first 2 days followed by a 750 mg QD for consecutive 5 days) was comparable among Chinese and non-Asian subjects. The mean accumulation ratios of C_max_ and AUC were 1.78 and 2.23, respectively in Chinese subjects. Similarly, the accumulation ratios of C_max_ and AUC were 2.11 and 2.54, respectively in non-Asian subjects overseas.

It is important to consider that the present study and phase 1 overseas studies were conducted with different populations and under different conditions, therefore, the comparison of PK parameters of ibrexafungerp should be interpreted with caution. The observed difference in AUC_0**−∞**_ might be partly related to the different drug distribution and clearance (CL). Owing to the fact that ibrexafungerp is highly protein bound (> 99%) (16), slight ethnic differences in protein binding may result in the dramatic changes in PK parameters (21). Mean apparent CL was larger (77.47 L/h) in this study (600 mg cohort) than in the previous study overseas (51.1 L/h). It is well known that genetic variations in metabolism of enzymes play a pivotal role in drug CL (22). CYP2C9, CYP2C19, and CYP2D6 being highly polymorphic are responsible for PK differences and drug response (23). But metabolism of ibrexafungerp is mainly through CYP3A4. As a result, genetic polymorphism of ibrexafungerp metabolism is unlikely to explain the lower AUC_0**−∞**_ in Chinese subjects. In addition, the variability of dietary habits and gut microbiota composition between two populations may also contribute to the CL (24, 25), because the majority of ibrexafungerp is excreted through faces.

### Conclusion

Following a single dose up to 1,500 mg and multiple doses up to 750 mg, ibrexafungerp was safe, well-tolerated, and displayed a favorable PK profile in healthy Chinese subjects, which was similar to those in the previous studies conducted overseas. These results support further clinical development of ibrexafungerp in China.

## Data Availability

The source data (containing the subjects personal information) that support the findings of this study are available from Jiangsu Hansoh Pharmaceutical Group Co., Ltd. Restrictions apply to the availability of these data, which were used under license for this study. Data are available from Shuwen Yu with the permission of Jiangsu Hansoh Pharmaceutical Group Co., Ltd.

## SUPPLEMENTAL MATERIAL

Supplemental file 1 aac.01075-23-s0001.docx Criteria for inclusion and exclusion and Tables S1 and S2.

ASM does not own the copyrights to Supplemental Material that may be linked to, or accessed through, an article. The authors have granted ASM a non-exclusive, world-wide license to publish the Supplemental Material files. Please contact the corresponding author directly for reuse.
