# Supplementary material for: Safety, tolerability, and pharmacokinetics of ibrexafungerp in healthy Chinese subjects: a randomized, double-blind, placebo-controlled phase 1 trial
Source: Antimicrob Agents Chemother. 2023 Nov 16;67(12):e01075-23. doi: 10.1128/aac.01075-23 (PMC10720486; doi:10.1128/aac.01075-23)
Supplement: Supplemental file 1 — Criteria for inclusion and exclusion and Tables S1 and S2. [file aac.01075-23-s0001.docx]

**SUPPLEMENTARY INFORMATION**

1. **Criteria for inclusion, exclusion, and stopping dose escalation of present phase 1 study in China.**

**1.1 Inclusion criteria**

1. Healthy subjects should be aged between 18 and 45 years old (critical values inclusive);
2. Subjects should have a full understanding of the contents and process of the trial and possible adverse reactions, and voluntarily sign the informed consent form (ICF);
3. A body mass index (BMI= weight[kg]/height^2^[m] ^2^) of 19~27 kg/m^2^ (critical values inclusive), and a body weight ≥ 50 kg for males and ≥ 45 kg for females at screening;
4. At screening, only subjects who have been assessed by the investigators to have no clinically significant abnormality (in terms of medical history inquiry, physical examination, vital signs (blood pressure, pulse, respiration, and body temperature), oxygen saturation (SpO_2_), laboratory tests (hematology, blood biochemistry, coagulation function and urine routine), ECG, abdominal B ultrasound, and Chest X ray-PA & LAT may be enrolled in this study;
5. Agree to take effective contraceptive measures from the date of signing the informed consent form to 90 days after the last dose (use of condoms, abstinence, vasectomy at least 3 months before the screening period, etc. for males; use of intrauterine devices, sexual partners’ use of male condoms, abstinence, etc. for females);
6. Female subjects have negative blood pregnancy tests at screening and baseline;
7. Male subjects agree to avoid donation of sperms from the start of administration until 90 days after the end of the study.

**1.2 Exclusion criteria**

1. Presence of clinically significant diseases at screening (such as nervous, mental, cardiovascular, urinary, digestive, respiratory, musculoskeletal, metabolic, endocrine, dermal, hematological and immune systems or tumors), which makes a subject inappropriate for participation in this study as assessed by the investigator;
2. Abnormalities in 12-lead ECG, which are judged as clinically significant by the investigator, e.g., based on the QT interval (QTcB) corrected by Bazett (formula: QT/RR^0.5^), absolute QTcB > 450 ms for males, and absolute QTcB > 470 ms for females;
3. Endogenous creatinine clearance < 80 mL/min at screening, calculated according to the Cockcroft-Gault formula: Endogenous creatinine clearance (mL/min) =$\frac{\left（ 140-Age \right）\times Body weight\left（ \mathrm{kg} \right）}{72\times Serum creatinine concentration\left（ \frac{\mathrm{mg}}{\mathrm{dL}} \right）}\left（ \mathrm{Female}\times0.85 \right）$;
4. Previous history of gastrointestinal diseases, including peptic ulcer, gastroesophageal reflux or gastrointestinal surgery, etc., which makes a subject inappropriate for participation in this study as assessed by the investigator;
5. Positive result in breath alcohol test or a history of alcoholism (consumption of more than 14 units of alcohol per week on average), or single use of more than 14 units of alcohol (1 unit = 285 ml of beer, 25 mL of spirit, or 125 mL of wine) in the past two weeks;
6. Any drugs and health care products, including prescription drugs, over-the-counter drugs and TCM drugs (including oral contraceptives, topical spermicides, drugs absorbed transdermally to produce systemic therapeutic effects and St. John's wort, etc.) taken within 2 weeks prior to screening or within 5 half-lives (whichever is longer) and expected to be taken throughout the study;
7. Daily consumption of more than six cups (approximately 250 mL per cup) of coffee, tea, coke, or other caffeinated beverages on average within 3 months prior to screening;
8. Consumption of foods that may affect drug metabolism, such as grapefruit juice, blood oranges, mulberry juice, mustard vegetables (such as kale, watercress, mustard, Brussels sprouts, and mustard greens) or charcoal roasted meat within 2 weeks prior to screening;
9. Significant blood loss or blood donation (> 250 mL) within 3 months prior to screening;
10. Those who have undergone major surgery in the past;
11. Subjects who have participated in any clinical trial or taken clinical trial drugs within 3 months prior to screening (except for subjects who have failed screening);
12. Those who have difficulties in swallowing capsules, tablets, or other solid preparations;
13. Those with a previous history of severe allergies to drugs, foods and substances (including allergies to latex, dust mites, pollen, etc.), or known allergy to any ingredient of the investigational drug;
14. Those with a history of drug dependence or abuse, or positive result in urine drug test at screening;
15. Those with daily smoking of more than five cigarettes within 3 months before this study, or inability to discontinue smoking of any tobacco products during this study;
16. Positive result in HBsAg, HCVAb, syphilis, or HIV antibody test for screening of infectious diseases at screening;
17. Dieting or dietary treatment for any cause, or a significant change in dietary habits within 2 weeks prior to screening.
18. Any physiological or mental disease or status that may increase risks in this study or affect subjects’ compliance with the protocol or ability to complete this study as assessed by the investigators;
19. Female subjects during pregnancy or lactating period;
20. Those having difficulties in blood collection, unable to bear multiple venous blood collections, or having any blood collection-related contraindications;
21. Those with a history of vaccination within 30 days prior to screening, or those with a vaccination schedule throughout the study.
    1. **Criteria for stopping dose escalation.**
22. Any subject in the dose group has a serious adverse event (SAE) and the SRC judges that the event is related to ibrexafungerp and may require termination of the current trial or permit continuation of the trial with a lower dose than the current dose, depending on the nature of the AEs;
23. If the number of subjects with any SAE in a dose group is ≥ 3 and the SRC judges that the event is related to ibrexafungerp, it may be necessary to terminate the current trial or continue the trial at a lower dose than the current dose;
24. The number of subjects with any SAE at the sentinel cohort of the second dose group at MAD stage is ≥ 1, and the researcher judge that the event is related to ibrexafungerp;
25. The number of subjects in the second dose group at MAD stage who experienced a SAE at the sentinel cohort is ≥ 1, and the researcher judge that the event is related to ibrexafungerp;
26. The number of subjects in the second dose group at the MAD stage who presented with aspartate aminotransferase (AST) or alanine aminotransferase (ALT) ≥ 3 times the upper limit of normal value and serum total bilirubin ≥ 2 times the upper limit of normal value at the sentinel cohort is ≥ 1;
27. In the second dose group at MAD stage, a 12-lead electrocardiogram showed that the absolute value of QTcB was > 500 ms, or the QTcB was longer than the baseline by > 60 ms, and the number of subjects confirmed by retest is ≥ 1;
28. If the number of subjects taking ibrexafungerp orally in a certain dose group experiences ≥ 2 cases of the following same adverse events, the SRC will not allow the dose to increase. However, if two subjects experience different adverse events, SRC will allow a dose lower than expected to increase. As follows:
    1. Systolic blood pressure increased by 60 mmHg, decreased by 30 mmHg, and systolic blood pressure ≤ 70 mmHg or ≥ 200 mmHg compared to before administration, lasting for at least 5 minutes;
    2. Diastolic blood pressure increased by 40 mmHg, decreased by 30 mmHg, and diastolic blood pressure ≤ 40 mmHg or ≥ 110 mmHg compared to before administration, lasting for at least 5 minutes;
    3. Heart rate increased by 50 bpm, decreased by 30 bpm, and absolute value ≤ 45 bpm or ≥ 170 bpm compared to before administration, lasting for at least 5 minutes;
    4. QT interval extension, defined as QTcB extension > 60 ms compared to the baseline, or QTcB absolute value > 500 ms;
    5. Repeated detection of ALT or AST within 48 hours > 5 x ULN;
    6. Repeated detection of total bilirubin within 48 hours > 2 x ULN;
    7. Repeated detection of ALT or AST within 48 hours > 2 x ULN with total bilirubin exceeding 1.5 x ULN;
    8. Repeated detection within 48 hours Cr > 1.5 x ULN;
    9. Repeated detection within 48 hours WBC < 2.0 x 10^9^/L;
    10. Repeated detection within 48 hours neutrophil count < 1.0 x 10^9^/L;
    11. Repeated detection within 48 hours platelet count < 50 x 10^9^/L.
29. **All treatment-emergent adverse events (TEAEs) in Single-ascending dose (SAD) and Multiple-ascending dose (MAD) stage of present phase 1 study in China were presented in Table S1 and Table S2.**

**Table S1. All treatment-emergent adverse events (TEAEs) in Single-ascending dose stage in China.**

|  | Placebo (N=6) n(%) | Ibrexafungerp | | | |
| --- | --- | --- | --- | --- | --- |
|  |  | **300mg (N=12) n(%)** | **600mg (N=12) n(%)** | **1500mg (N=12) n(%)** | **Total (N=36) n(%)** |
| All AEs | 3(50.0) | 8(66.7) | 11(91.7) | 12(100.0) | 31(86.1) |
| Gastrointestinal disorders | 1(16.7) | 5(41.7) | 8(66.7) | 12(100.0) | 25(69.4) |
| Diarrhea, | 1(16.7) | 5(41.7) | 8(66.7) | 12(100.0) | 25(69.4) |
| Abdominal pain | 0 | 5(41.7) | 1(8.3) | 7(58.3) | 13(36.1) |
| Vomiting | 0 | 0 | 0 | 4(33.3) | 4(11.1) |
| Nausea | 0 | 0 | 0 | 3(25.0) | 3(8.3) |
| Abdominal bloating | 0 | 0 | 1(8.3) | 0 | 1(2.8) |
| Laboratory abnormalities | 1(16.7) | 5(41.7) | 7(58.3) | 6(50.0) | 18(50.0) |
| Hemoglobin decreased | 0 | 0 | 2(16.7) | 2(16.7) | 4(11.1) |
| Blood triglycerides increased | 1(16.7) | 2(16.7) | 0 | 1(8.3) | 3(8.3) |
| Blood pressure increased | 0 | 3(25.0) | 0 | 0 | 3(8.3) |
| Urine erythrocyte positive | 0 | 0 | 1(8.3) | 1(8.3) | 2(5.6) |
| Urine occult blood positive | 0 | 0 | 0 | 2(16.7) | 2(5.6) |
| Presence of urinary ketones | 0 | 0 | 0 | 2(16.7) | 2(5.6) |
| Uric acid increased | 0 | 0 | 1(8.3) | 1(8.3) | 2(5.6) |
| White blood cell increased | 0 | 0 | 1(8.3) | 0 | 1(2.8) |
| LDL increased | 0 | 0 | 0 | 1(8.3) | 1(2.8) |
| Prolonged thrombin time | 0 | 0 | 0 | 1(8.3) | 1(2.8) |
| ECG ST segment elevated | 0 | 0 | 1(8.3) | 0 | 1(2.8) |
| Abnormal ECG T-wave | 0 | 1(8.3) | 0 | 0 | 1(2.8) |
| Heart rate decreased | 0 | 0 | 1(8.3) | 0 | 1(2.8) |
| Blood bilirubin increased | 0 | 0 | 1(8.3) | 0 | 1(2.8) |
| Blood creatine kinase increased | 0 | 1(8.3) | 0 | 0 | 1(2.8) |
| Vascular and lymphatic diseases | 1(16.7) | 2(16.7) | 2(16.7) | 0 | 4(11.1) |
| Orthostatic hypotension | 1(16.7) | 2(16.7) | 2(16.7) | 0 | 4(11.1) |
| Neurological diseases | 1(16.7) | 0 | 0 | 1(8.3) | 1(2.8) |
| Dizziness | 0 | 0 | 0 | 1(8.3) | 1(2.8) |
| Syncope | 1(16.7) | 0 | 0 | 1(8.3) | 1(2.8) |
| Heart diseases | 1(16.7) | 0 | 1(8.3) | 0 | 1(2.8) |
| Sinoatrial block | 0 | 0 | 1(8.3) | 0 | 1(2.8) |
| Ventricular extrasystole | 1(16.7) | 0 | 0 | 0 | 0 |

**Table S2. All treatment-emergent adverse events (TEAEs) in Multiple-ascending dose stage in China.**

|  | Placebo  (N=4) n(%) | Ibrexafungerp | | | |
| --- | --- | --- | --- | --- | --- |
|  |  | **450mg (N=12) n(%)** | **750mg (N=12) n(%)** | | **Total (N=24) n(%)** |
| All TEAEs | 3(75.0) | 12(100.0) | 12(100.0) | 24(100.0) | |
| Gastrointestinal disorders | 1(25.0) | 10(83.3) | 12(100.0) | 22(91.7) | |
| Diarrhea | 0 | 10(83.3) | 12(100.0) | 22(91.7) | |
| Abdominal pain | 1(25.0) | 5(41.7) | 10(83.3) | 15(62.5) | |
| Nausea | 0 | 2(16.7) | 6(50.0) | 8(33.3) | |
| Vomiting | 0 | 0 | 6(50.0) | 6(25.0) | |
| Gastroesophageal reflux disease | 0 | 0 | 2(16.7) | 2(8.3) | |
| Constipation | 0 | 1(8.3) | 0 | 1(4.2) | |
| Abdominal bloating | 0 | 0 | 1(8.3) | 1(4.2) | |
| Oral mucositis | 0 | 0 | 1(8.3) | 1(4.2) | |
| Laboratory abnormalities | 1(25.0) | 5(41.7) | 8(66.7) | 13(54.2) | |
| Blood bilirubin increased | 0 | 2(16.7) | 3(25.0) | 5(20.8) | |
| ALT increased | 0 | 3(25.0) | 0 | 3(12.5) | |
| Blood pressure increased | 0 | 1(8.3) | 2(16.7) | 3(12.5) | |
| White blood cell increased | 0 | 0 | 2(16.7) | 2(8.3) | |
| Urine occult blood positive | 0 | 0 | 2(16.7) | 2(8.3) | |
| Aspartate aminotransferase increased | 0 | 2(16.7) | 0 | 2(8.3) | |
| Blood triglyceride increased | 1(25.0) | 2(16.7) | 0 | 2(8.3) | |
| Abnormal ECG T-wave | 0 | 0 | 1(8.3) | 1(4.2) | |
| Blood creatine kinase increased | 0 | 0 | 1(8.3) | 1(4.2) | |
| Metabolic and nutritional diseases | 0 | 1(8.3) | 3(25.0) | 4(16.7) | |
| Hypertriglyceridemia | 0 | 0 | 3(25.0) | 3(12.5) | |
| Hyperglycemia | 0 | 1(8.3) | 0 | 1(4.2) | |
| Heart diseases | 1(25.0) | 1(8.3) | 2(16.7) | 3(12.5) | |
| Tachycardia | 0 | 1(8.3) | 2(16.7) | 3(12.5) | |
| Bradycardia | 1(25.0) | 0 | 0 | 0 | |
| Infectious and infective diseases | 0 | 0 | 2(16.7) | 2(8.3) | |
| Urinary tract infection | 0 | 0 | 2(16.7) | 2(8.3) | |
| Neurological diseases | 0 | 0 | 2(16.7) | 2(8.3) | |
| Dizziness | 0 | 0 | 2(16.7) | 2(8.3) | |
| Diseases of the skin and subcutaneous tissue | 0 | 0 | 1(8.3) | 1(4.2) | |
| Maculopapulae | 0 | 0 | 1(8.3) | 1(4.2) | |
| Systemic disease and various reactions at the site of administration | 0 | 1(8.3) | 0 | 1(4.2) | |
| Chest discomfort | 0 | 1(8.3) | 0 | 1(4.2) | |
| Vascular and lymphatic diseases | 0 | 0 | 1(8.3) | 1(4.2) | |
| Orthostatic hypotension | 0 | 0 | 1(8.3) | 1(4.2) | |
